# Damage-Accumulation-Induced Crack Propagation and Fatigue Life Analysis of a Porous LY12 Aluminum Alloy Plate

**DOI:** 10.3390/ma17010192

**Published:** 2023-12-29

**Authors:** Cheng Lv, Kejie Wang, Xiang Zhao, Fenghui Wang

**Affiliations:** 1 Bio-Inspired and Advanced Energy Research Center, School of Mechanics, Civil Engineering and Architecture, Northwestern Polytechnical University, Xi’an 710129, China; 2 Aircraft Strength Research Institute of China, Xi’an 710065, China

**Keywords:** crack propagation, fatigue life calculation, porous structure, extended finite element method

## Abstract

Rivets are usually used to connect the skin of an aircraft with joints such as frames and stringers, so the skin of the connection part is a porous structure. During the service of the aircraft, cracks appear in some difficult-to-detect parts of the skin porous structure, which causes great difficulties in the service life prediction and health monitoring of the aircraft. In this paper, a secondary development subroutine in PYTHON based on ABAQUS-XFEM is compiled to analyze the cracks that are difficult to monitor in the porous structure of aircraft skin joints. The program can automatically analyze the stress intensity factor of the crack tip with different lengths in the porous structure, and then the residual fatigue life can be deduced. For the sake of safety, the program adopts a more conservative algorithm. In comparison with the physical fatigue test results, the fatigue life of the simulation results is 16% smaller. This project provides a feasible simulation method for fatigue life prediction of porous structures. It lays a foundation for the subsequent establishment of digital twins for damage monitoring of aircraft porous structures.

## 1. Introduction

Cracks in porous structures are a significant contributor to widespread fatigue damage in aircraft. Extensive experimental studies have demonstrated that the fatigue life of a porous collinear crack structure is substantially shorter than that of a single-hole single crack. As a result, the presence of multihole collinear cracks is a crucial factor influencing the prediction and assessment of aircraft fatigue life.

For the study of crack propagation in porous structures, the crack stress intensity factor (SIF) should be obtained first. At present, the stress intensity factor methods for different structures are as follows: The SIF approximate analytical solution of a typical porous multicrack problem can be obtained using the Eshelby inclusion theory and the weight function method [1]. Using the finite element calculation model, a variety of porous multicrack SIFs can be obtained [2]. The SIF of the multiple site damage (MSD) of porous structures is solved by the boundary correction factor combination method [3]. The boundary-based finite element method (BFEM) is used to deal with two-dimensional anisotropic elastic solid problems with multiple holes and cracks. The SIF is calculated with the newly revised formula [4]. For a variety of different porous multicrack structures, the composite method is used to evaluate SIF by superimposing a set of appropriate elementary solutions to consider different geometric boundaries [5]. The integral mean is used to quantify the SIF at the crack tip [6]. The multicrack SIF is obtained with the prediction–correction method combined with the improved virtual crack closure integral (MVCCI) method [7]. Based on the Cauchy integral method in complex variable theory, Rao established a new integral equation method to solve the SIF of the interaction of anisotropic elastic solids with multiple elliptical holes and cracks under remote stress and nonuniform surface stress [8].

For the study of fatigue crack propagation, there are, mainly, the following methods. By studying the phase field method, based on the variational method of damage location, the complex crack trajectory is described [9]. The interaction between multiple cracks and crack propagation is studied using the boundary element method [10]. The complex variable function is used to derive the stress function, and the complex multipoint damage (MSD) problem can be transformed into a simple single-point damage problem by using the approximate superposition method [11]. Through multiple sets of fatigue tests and the Wiener process of failure mechanisms, a method to describe the random fatigue crack propagation process is obtained [12]. Seifi conducted finite element method simulation tests and physical experiments on hollow prenotched plates with multipoint damage (MSD) to evaluate the effects of parameters such as thickness, aperture, and hole center distance on crack propagation [13]. Wang theoretically studied the multipoint damage (MSD) crack propagation behavior in mechanically fastened fiber metal laminate (FML) joints and modeled the effects of bearing and bypass loads. The model can successfully predict the rapid crack propagation near the fastener hole caused by the interaction of bearing stress and the crack [14]. Cui used a finite element model of centerline cracks under small-scale yield conditions to study the effect of interaction between holes in front of the crack on the ductile fracture process [15]. Chang studied the crack propagation of porous hybrid S2-glass-reinforced aluminum laminates with multipoint damage (MSD). Based on the empirical Paris formula, the stress intensity factor is calculated, and an analytical crack growth model for predicting the crack growth rate is established to predict the trend of MSD average crack growth [16]. Ambriz studied the effect of the variable load spectrum on fatigue crack growth (FCG) [17]. Doan established the vibration equation using the finite element method to simulate the free vibration response of the cracked nanoplate, considering the flexoelectric effect [18]. Cong used the modified couple stress theory and the phase field theory to study the crack propagation behavior of plane strain microplates [19]. Based on a complete mathematical model, Truong Anh established a von Mises strength model using the highly nonlinear dynamics software AUTODYN to solve the problem of metal plate damage in contact with an explosive charge [20]. Van Do used the phase field model to study the thermal buckling of functionally graded material (FGM) plates with cracks [21]. Based on the improved Paris model and particle filter (PF) framework, Wang proposed a new multifatigue crack growth prediction method to predict metal multicracks. By introducing the coupling interaction of multiple cracks in the SIF expression, the modified Paris formula is used to predict the propagation of multiple fatigue cracks. In addition, the PF framework is used to combine the prediction results of the modified Paris model with the measurement results of the online monitoring model to reduce the uncertainty of material parameters and obtain more accurate prediction results [22]. Lv wrote the secondary development extended finite element program of the finite element software Abaqus in the Python language to simulate the fatigue crack propagation of the aluminum plate [23].

For porous collinear cracks, the boundary finite element method is suitable for the general case of porous collinear cracks [24]. Xu applied the weight function method to study the cracks in the collinear side holes of aircraft structures and any small cracks generated by multiple collinear holes in infinite sheets and gave an explicit closed-form weight function. An approximate weight function method is proposed to calculate the stress intensity factor of small cracks in collinear holes, which is more efficient than the finite element method [25]. Moreover, the initiation point and propagation rate of multihole collinear cracks can be studied by examining the fracture surface [26]. For porous collinear cracks, when the crack does not penetrate a hole, the hole is equivalent to a crack stop hole. At present, most of them are based on finite element simulation to study the repair effect of different crack arrest hole arrangements [27] and optimize the parameters of crack arrest holes to improve the crack arrest effect [28]. Malekan studied the effects of arresting holes and multiple microcracks and elliptical holes with different characteristic lengths and geometries on fatigue crack growth of aluminum alloy plates using the extended finite element method [29]. Ahmed used the boundary crack method (BCM) to simulate the porous multicrack plate and predicted the crack propagation path and fatigue life [30]. Bhatia studied the effects of different layouts of the two holes and the layout and size of different repair plates on the fatigue life of the repaired laminates [31].

In this paper, based on the PYTHON secondary development subroutine of ABAQUS-XFEM, the strength factor and crack propagation process of different lengths of cracks in the porous structure are automatically calculated, and then the residual fatigue life of the structure is derived. By comparing with the results of a physical fatigue test, the safety and effectiveness of the method are verified, which provides a method for the subsequent establishment of digital twins for damage monitoring of aircraft porous structures.

## 2. Stress Intensity Factor and Fatigue Crack Propagation Theory

### 2.1. Stress Intensity Factor

**Figure 1:**
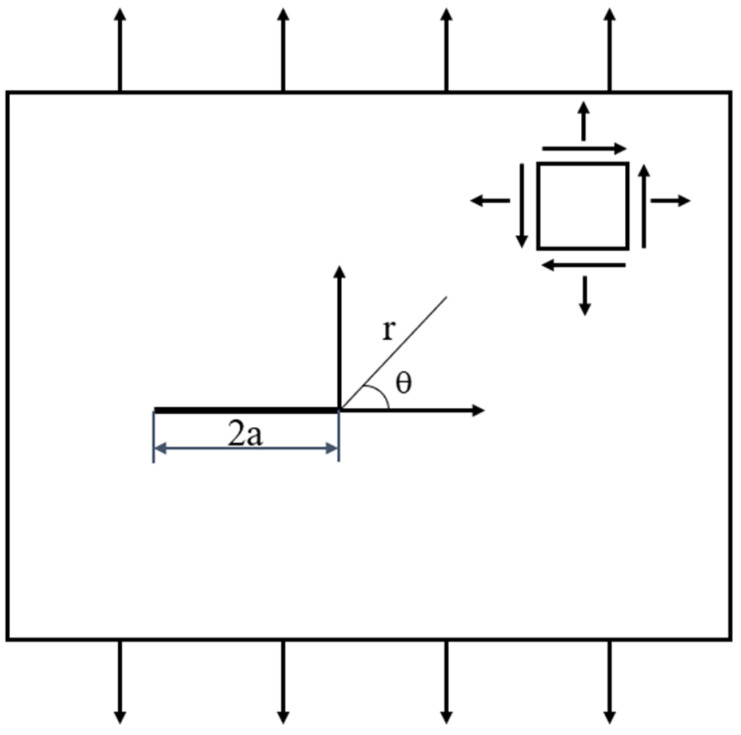
Stress boundary conditions of mode I crack under uniaxial tension.

The stress boundary conditions of the mode I crack under uniaxial tension are shown in Figure 1.

The stress field in the vicinity of the crack tip (r → 0) under uniaxial tension is shown in Equations (1)–(3):

(1) $$\sigma_{x}=\frac{K_{I}}{\sqrt{2\pi r}}\cos\theta [1-\sin\frac{\theta }{2}\sin\frac{3\theta }{2}]+O(r^{1/2})$$

(2) $$\sigma_{y}=\frac{K_{I}}{\sqrt{2\pi r}}\cos\theta [1+\sin\frac{\theta }{2}\sin\frac{3\theta }{2}]+O(r^{1/2})$$

(3) $$\tau_{xy}=\frac{K_{I}}{\sqrt{2\pi r}}\sin\frac{\theta }{2}\cos\frac{\theta }{2}\cos\frac{3\theta }{2}+O(r^{1/2})$$

$O(r^{1/2})$ is the stress field near the high-order fatigue crack tip that changes with the coordinate. When the crack type is given, the stress field distribution can be determined. The stress near the crack tip is singular. In engineering practice, the stress singularity at the crack tip will produce relaxation caused by plasticity due to the limitation of material yield. The stress intensity factor is independent of the coordinates of the crack tip, which mainly reflects the stress intensity near the fatigue crack tip. The value of the stress intensity factor is mainly determined by the size of the applied external load, the loading method, and the geometric shape of the cracked body. The specific expression is shown in Formula (4):

(4) $$K_{I}=\beta \sigma \sqrt{\pi a}$$

In the formula, $K_{I}$ is the stress intensity factor, σ is the applied stress, and a is the fatigue crack length. The stress intensity factor correction factor is a dimensionless function related to the crack shape, load-loading method, geometric parameters of the cracked body, and other factors.

For the extended finite element method, we can obtain the stress value at the crack tip through simulation analysis and then calculate the stress intensity factor value through Formulas (1)–(3).

For the crack tip stress of mode II (sliding mode) subjected to uniform pure shear stress, calculations can be performed as shown in Formulas (5)–(7):

(5) $$\sigma_{x}=\frac{-K_{II}}{\sqrt{2\pi r}}\sin\frac{\theta }{2}[2+\cos\frac{\theta }{2}\cos\frac{3\theta }{2}]+O(r^{1/2})$$

(6) $$\sigma_{y}=\frac{K_{II}}{\sqrt{2\pi r}}\sin\frac{\theta }{2}\cos\frac{\theta }{2}\cos\frac{3\theta }{2}+O(r^{1/2})$$

(7) $$\tau_{xy}=\frac{K_{II}}{\sqrt{2\pi r}}\cos\frac{\theta }{2}[1-\sin\frac{\theta }{2}\sin\frac{3\theta }{2}]+O(r^{1/2})$$

In the formula

(8) $$K_{II}=\beta \tau \sqrt{\pi a}$$

For the extended finite element method, we can obtain the stress value at the crack tip through simulation analysis and then calculate the stress intensity factor value using Formulas (5)–(7).

### 2.2. Fatigue Crack Propagation Analysis Model

The Walker formula is based on the Paris formula and considers the influence of the stress ratio on the crack propagation life, as shown in Formula (9):

(9) $$da/dN=C{\left(Z\cdot K_{\max}\right)}^{n}$$

The meaning of each parameter is as follows:

Maximum stress intensity factor $K_{\max}$:

(10) $$K_{\max}=\beta \cdot \sigma_{\max}\sqrt{\pi a}$$

In the formula, $\sigma_{\max}$ is the maximum working principal stress, β is the comprehensive correction factor, and a is the crack length under the cracking mode.

Minimum stress factor Z:

(11) $$Z=\left\{\begin{array}{l} {\left(1-R\right)}^{m},0<R<1.0\\ {\left(1-R\right)}^{q},R\leq 0\\ 0,R=1.0 \end{array}\right\}$$

In the formula, m and q are the expansion performance parameters of the material, and R is the stress ratio.

This is compared with ignoring the influence of the loading sequence under constant amplitude load. The Willenborg–Chang model considers the effect of variable amplitude load based on the improved Walker formula. An effective stress ratio is introduced to replace the nominal stress ratio $R_{eff}$, which mainly considers the influence of the loading sequence on the expansion rate.

(12) $$da/dN=C{\left(\frac{\Delta K}{{\left(1-R_{eff}\right)}^{1-m}}\right)}^{n}$$

## 3. Fatigue Crack Propagation Simulation Based on XFEM

### 3.1. The Calculation of the Stress Intensity Factor Based on ABAQUS-XFEM

**Figure 2:**
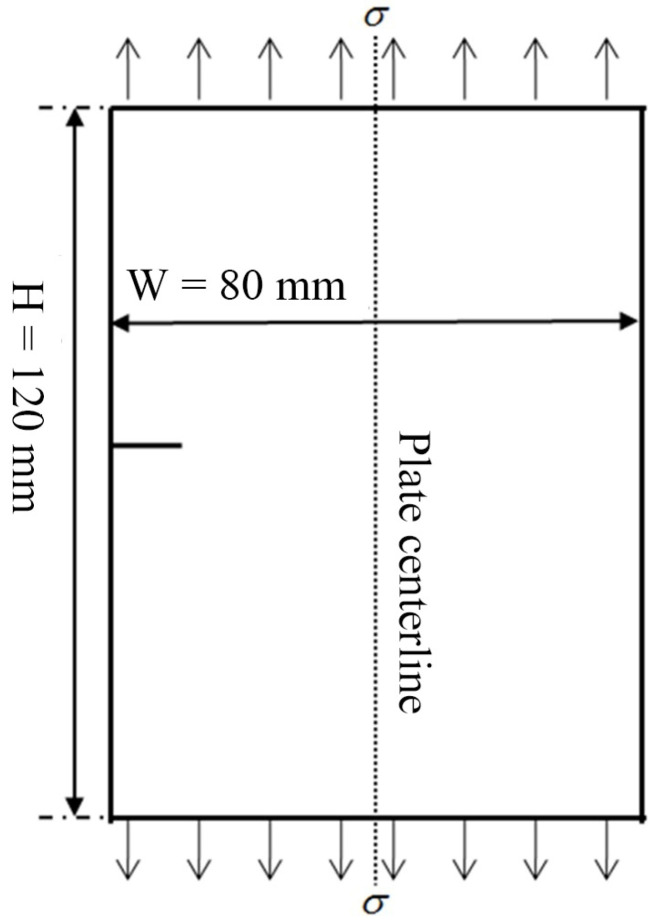
Edge-penetrating crack model of finite-width plate.

The finite element analysis model shown in Figure 2 is established. The crack is located at the left edge of the thin plate and uniformly loaded at both ends.

**Figure 3:**
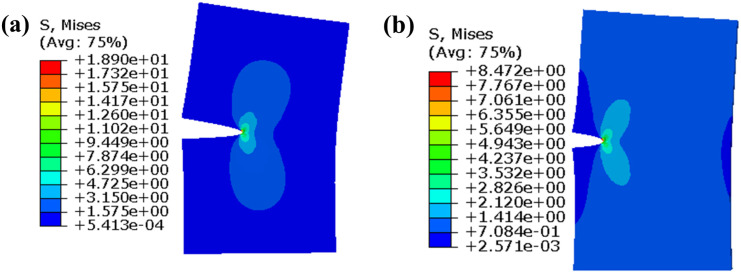
Stress cloud diagram of type I crack propagation finite element model with (**a**) crack length a = 16 mm; (**b**) crack length a = 8 mm.

The stress cloud diagram of the corresponding extended finite element model with different crack lengths is shown in Figure 3.

The stress intensity factor at the crack tip is calculated as shown in Table 1. The calculated results are compared with the analytical models of finite width, edge-penetrating crack, and far uniform tensile load. The stress intensity factor of the engineering algorithm is shown in Formula (13):

(13) $$K=\sigma \sqrt{\pi a}g(\xi )$$

(14) $$\begin{array}{l} g(\xi )=1.12-0.231\xi +10.55\xi^{2}-\\ 21.72\xi^{3}+30.39\xi^{4} \end{array}$$

(15) ξ=a/W

The calculation results are shown in Table 1. The maximum error of the stress intensity factor calculation results of the crack lengths of 8 mm and 16 mm is about 3%.

### 3.2. Fatigue Crack Propagation Life Analysis Example Based on ABAQUS-XFEM

After determining the method of calculating the stress intensity factor at the crack tip and the fatigue crack growth rate, the fatigue crack growth life can be estimated. The PYTHON language subroutine based on ABAQUS-XFEM is compiled, which combines the calculation of the stress intensity factor and the calculation of the fatigue crack growth rate and realizes the calculation of the fatigue crack growth life of cracked structures.

**Figure 4:**
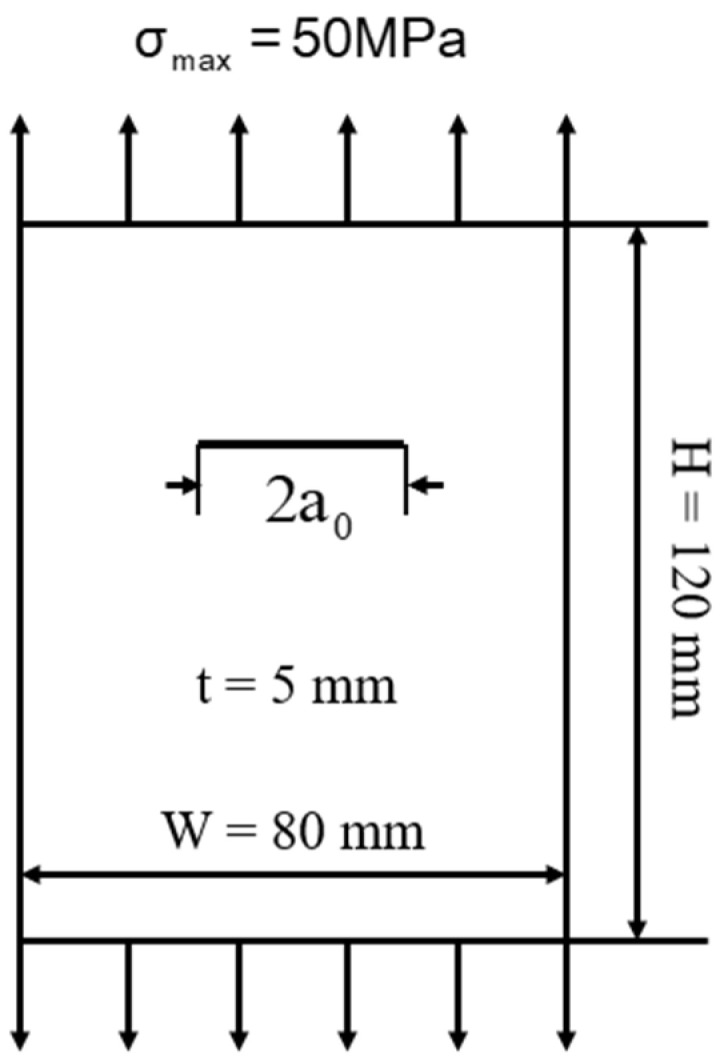
Crack propagation analysis model of center-cracked plate with finite width.

The analytical model shown in Figure 4 is established. The crack propagation life from 8 mm to 32 mm is calculated with the engineering algorithm and extended finite element method, respectively.

The cracking mode of the flat plate is the linear cracking mode. The Walker formula is used to calculate the crack propagation life. The material properties related to the crack propagation rate are $C=2.34\times 10^{-12}$, n=3.2, m=0.6.

(1) The engineering algorithm to calculate crack propagation life is as follows:

The number of cycles of crack propagation life is calculated using the variable separation method:

(16) $$N_{f}={\left[\frac{G_{C}}{\sigma }\right]}^{n}={\left[\frac{1740}{50}\right]}^{3.2}=85714.36$$

The geometric material factor is

(17) $$G_{C}={\left[C^{-1}\int_{a_{0}}^{a_{f}}{\left(\beta \sqrt{\pi a}\right)}^{-n}da\right]}^{1/n}=1740$$

The correction factor formula is

(18) $$\beta =\sqrt{\sec(\frac{\pi a}{W})}$$

**Figure 5:**
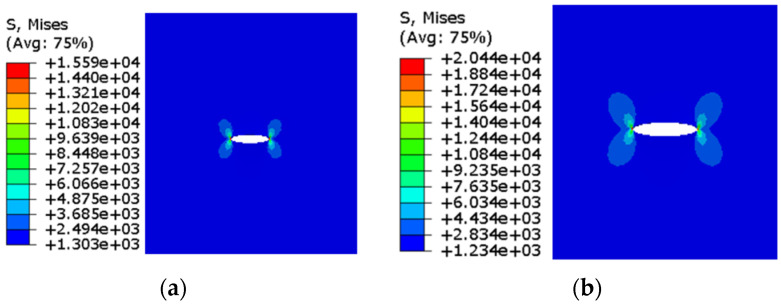
Crack propagation stress cloud diagram under different crack lengths with (**a**) crack length 2a = 15 mm; (**b**) crack length 2a = 24 mm.

(2) The extended finite element method to calculate crack propagation life is as follows:

The stress cloud diagram of crack propagation under different crack lengths is shown in Figure 5 (the amplification factor is 100).

**Figure 6:**
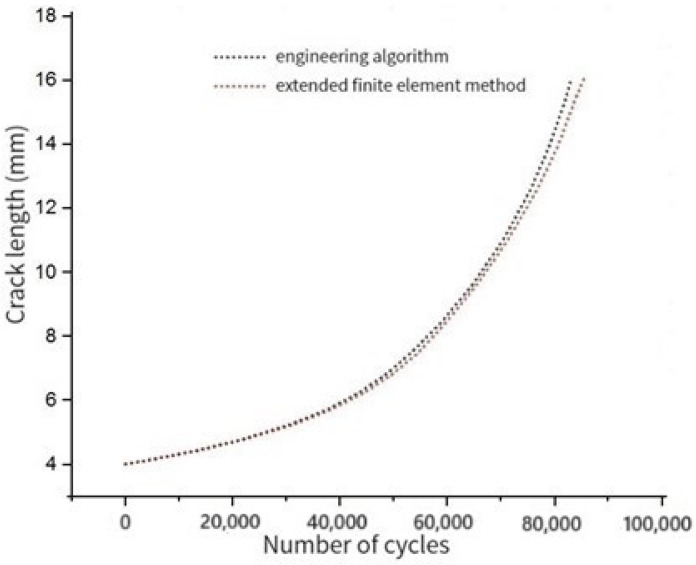
Crack length–cycle number curve.

The crack propagation life comparison results of the engineering algorithm and the extended finite element method are shown in Figure 6.

It can be seen that the crack propagation life analysis curves of the extended finite element method and the engineering algorithm are basically consistent.

## 4. Test and Simulation of Structural Fatigue Life Analysis Considering Damage Accumulation

### 4.1. Simplified Analysis Model of Fatigue Life Example Considering Damage Accumulation

#### 4.1.1. Establishment of Analysis Model

**Figure 7:**
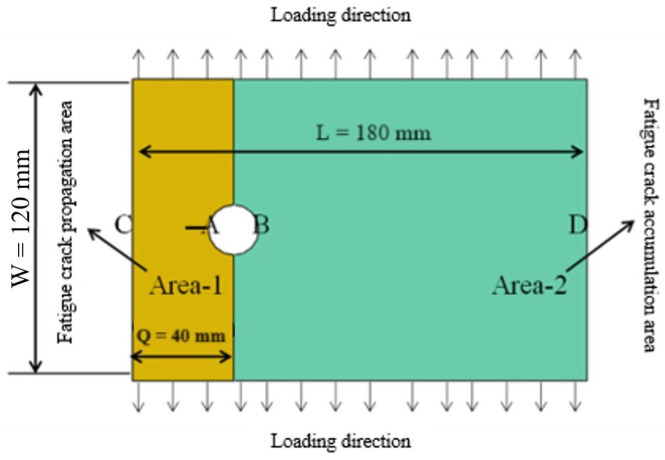
Crack propagation analysis model considering damage accumulation.

The analysis model shown in Figure 7 is established, and the initial state is assumed to be as follows: there is an initial crack of 5 mm at hole A, and the initial damage at hole B is zero. The crack propagation life is calculated in two stages. In the first stage, calculate the crack propagation life when the initial crack with a length of 5 mm from A extends to edge C, and calculate the fatigue damage accumulation at hole B at the same time. In the second stage, the crack has penetrated the AC, and the life of the initial crack at hole B due to damage accumulation through to the failure of the entire analysis structure is calculated.

The first stage analysis method of crack propagation is as follows: With the loading of fatigue load, the fatigue crack propagation life at hole A is calculated with the fracture mechanics crack propagation method, and the damage accumulation at hole B is calculated with the damage mechanics method. In other words, the crack propagation and damage accumulation are considered in the analysis process, and the fracture mechanics and damage mechanics are effectively combined. The main realization method is to set up two different materials, which are described as follows:

The material type in the crack propagation zone (as shown in Figure 7, ‘Area-1’) does not consider the cumulative fatigue damage. The main reason is that fracture mechanics and fatigue damage theory are two mutually analytical life theories.

In the process of crack propagation, there will be damage at hole B in the area ‘Area-2’ (as shown in Figure 7). At this time, the damage variable is added to the material properties of the area ‘Area-2’, and the USDFLD subroutine based on the fatigue damage evolution equation is written to realize the damage accumulation of the ‘damage accumulation area’ in the process of crack propagation and realize the whole life analysis of crack propagation.

#### 4.1.2. Fatigue Damage Evolution Equation

In 1958, L.M. Kachanov first proposed the introduction of continuity to reflect the continuous deterioration of materials. He believed that the deterioration mechanism of materials was the reduction of the effective bearing area caused by microdefects.

**Figure 8:**
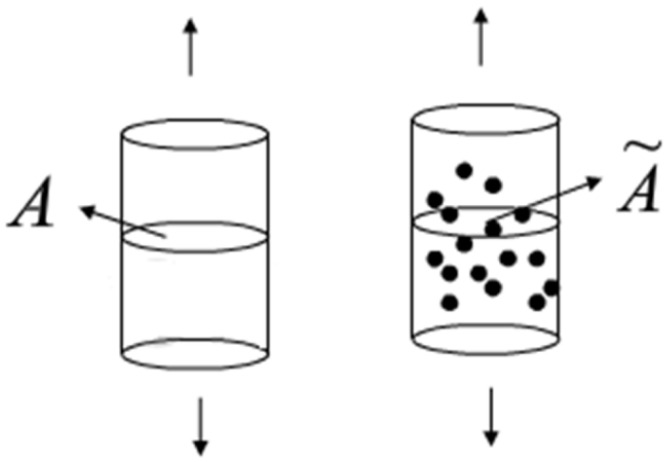
Damage diagram of uniaxial tensile body.

For the one-dimensional stretching case shown in Figure 8, the continuity ψ is shown in Formula (19).

(19) $$\psi =\frac{\overset{\sim }{A}}{A}$$

A is the apparent bearing section area of the material in the nondamaged state, and $\tilde{A}$ is the effective bearing section area in the damaged state.

ψ=1 indicates that the material state is an ideal state without any defects, and ψ=0 indicates that the material state is completely destroyed. When ψ reaches a critical value, the material is destroyed, and the failure criterion is

(20) $$\psi =\psi_{c}$$

Rabotnov, Y.N. extended the above concept, and the damage degree was proposed to describe the damage:

(21) $$D=1-\psi =\frac{A-\tilde{A}}{A}=\frac{A_{D}}{A}$$

The damage variable expressed by elastic modulus is shown in Formula (22):

(22) $$D=1-\frac{E_{D}}{E}$$

$E_{D}$ is the elastic modulus of the damaged material.

For fatigue damage analysis, the damage evolution equation of isotropic linear elastic damage material under fatigue load is shown in Formula (23):

(23) $$\frac{dD}{dN}=\frac{\alpha }{p+1}{\left(\frac{1}{2E}\right)}^{p+1}\frac{1}{{\left(1-D\right)}^{2p+2}}[\sigma_{e,max}^{2p+2}-\sigma_{th}^{2p+2}]$$

Among them, $\sigma_{e,max}$ is the equivalent stress corresponding to the maximum load of the material, α,p are the damage parameters, and $\sigma_{th}$ is the damage threshold stress of the material.

#### 4.1.3. Finite Element Simulation of Fatigue Life Analysis

**Figure 9:**
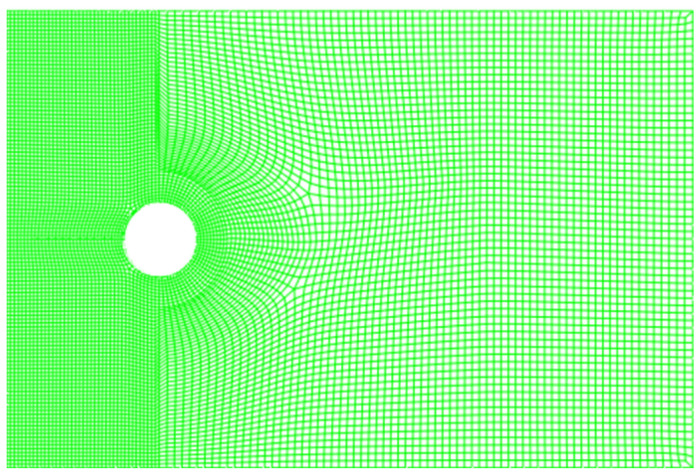
Analysis model meshing (the green line is a grid line, which is closer to the area that needs to be analyzed more intensively).

The material of the analysis model is LY12-CZ, and its material properties are shown in Table 2. The upper and lower surfaces are subjected to uniformly distributed equal amplitude loads, $S_{\max}=60\;\mathrm{MPa}$, $S_{\min}=20\;\mathrm{MPa}$. The finite element simulation mesh is shown in Figure 9, and the crack propagation zone and hole edge are refined.

Since the analysis model is a constant amplitude fatigue load, the crack growth rate formula uses the Walker formula to calculate the crack growth life, where the stress ratio and the material crack growth rate are related parameters. In addition, the stress intensity factor is calculated by the extended finite element method, and the fatigue damage is accumulated according to the fatigue damage evolution equation.

The finite element simulation results of the first stage of crack propagation are as follows: when the cycle life n = 68,628 times, the crack at hole A extends to 23.31 mm, the crack is unstable, the stress intensity factor reaches the critical value $K_{IC}=23.2\;\mathrm{MPa}\sqrt{m}$, and the damage value at hole B reaches D = 0.297.

**Figure 10:**
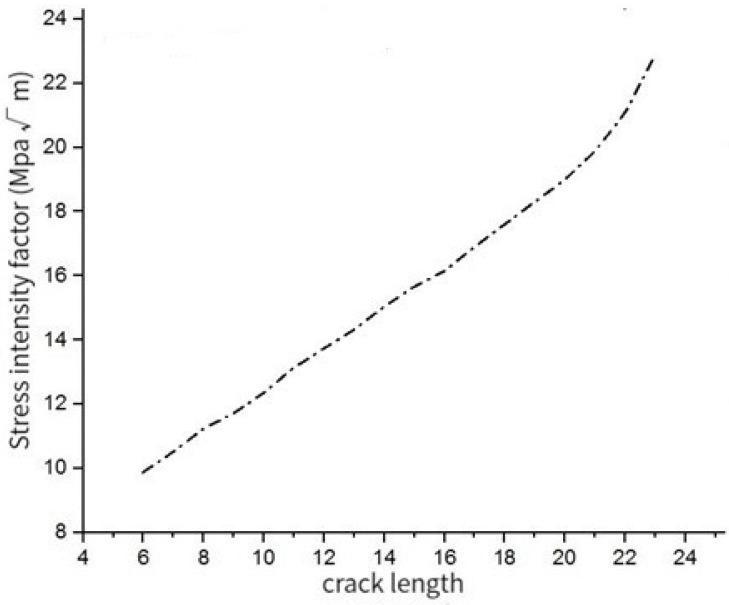
Curve of stress intensity factor with crack propagation length.

According to the finite element simulation results, the stress intensity factor *K_I_* at hole A changes with the crack propagation length, as shown in Figure 10.

With the continuous loading of fatigue load, the crack at hole A grows. When the crack length is less than 20 mm, the stress intensity factor KI increases at a stable rate. When the crack length is greater than 20 mm, the growth rate of stress intensity factor *K_I_* increases continuously and reaches the critical value of the stress intensity factor at a faster rate.

**Figure 11:**
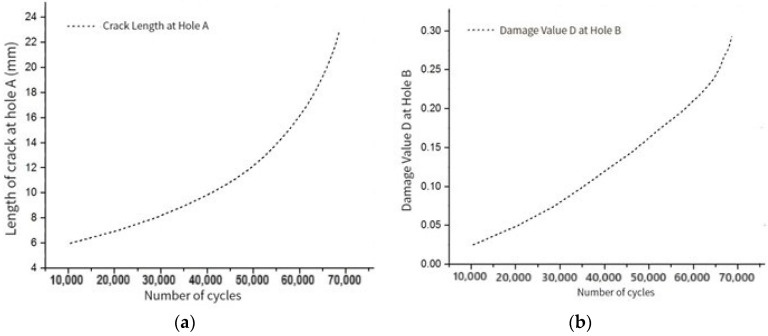
Finite element simulation results of the first stage of crack propagation with (**a**) crack length–cycle number curve at hole A; and (**b**) D-cycle curve of damage accumulation value at hole B.

According to the finite element simulation results, the number of fatigue cycles in the first stage of crack propagation, the crack length at hole A, and the damage accumulation at hole B are shown in Figure 11.

From the analysis of Figure 11, we see that the basic growth trend of the two curves is the same. With the loading of fatigue load, the crack length at hole A and the maximum damage at hole B initially increase at a stable rate. After the number of cycles reaches a certain stage, the crack length at hole A, and the maximum damage at hole B, the growth rate increases continuously.

According to the finite element simulation results, in the first stage of crack propagation, with the increase in the number of cycles, the crack length at hole A increases and the damage accumulation distribution cloud map near hole B is shown in Table 3.

**Figure 12:**
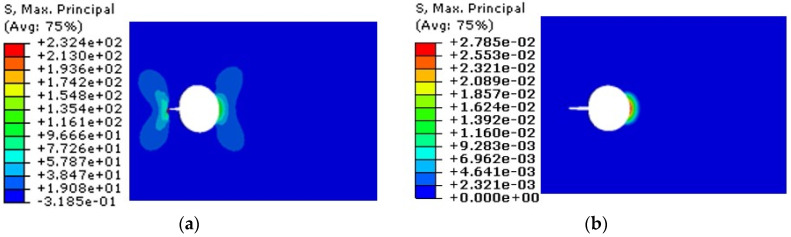
When the number of cyclic loading is N = 10,356, the stress cloud diagram and damage distribution diagram of the model are analyzed. (**a**) Stress cloud, and (**b**) damage distribution.

Among them, when the number of cyclic loading is N = 10,356, the crack length at hole A is 6 mm, and the maximum damage at hole B is D = 2.78 × 10^−2^. The stress cloud diagram and damage distribution diagram of the overall structure are shown in Figure 12.

### 4.2. Damage Fatigue Tolerance Test and Finite Element Simulation of Skin Porous Structure

#### 4.2.1. Damage Tolerance Test Equipment and Test Methods

**Figure 13:**
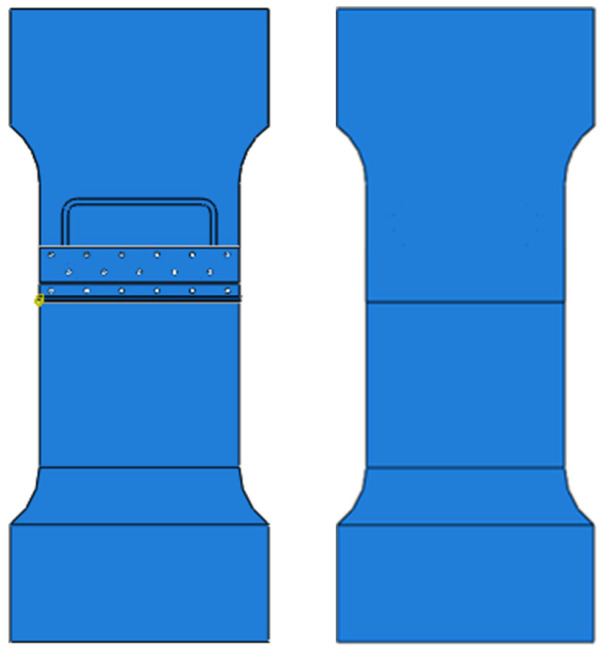
Fatigue damage tolerance test piece.

The fatigue damage tolerance test of the test piece shown in Figure 13 is carried out. The test piece is a typical aircraft stringer connector. Through the damage tolerance test of two groups of test pieces, the fatigue life of each test piece and the load-loading times under the corresponding fatigue crack length are obtained.

**Figure 14:**
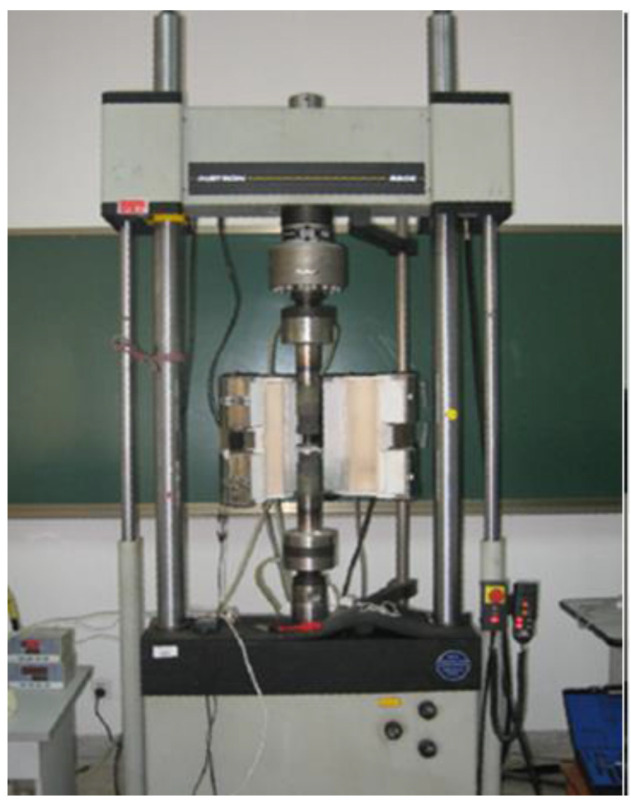
SDM100 electrohydraulic servo fatigue test machine.

The fatigue damage tolerance test equipment is shown in Figure 14.

The loading accuracy of the machine is 1%, which meets the requirements of fatigue testing.

#### 4.2.2. Test Results and Crack Propagation Path Analysis

The loading load and test results of each test piece are shown in Table 4.

**Figure 15:**
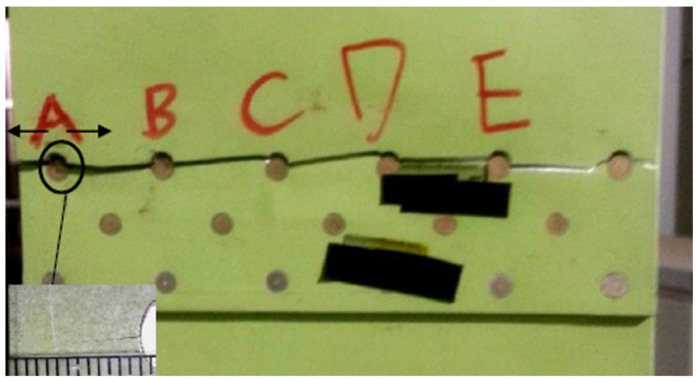
Fatigue crack initiation and fracture path of No. 1 test piece.

The fracture paths of each test piece are as follows:

The fatigue crack of the No. 1 test piece initiates from the boundary of the A rivet on the top right of the back of the test piece and expands to the B rivet. When it expands to the right to about 5.2 mm, fatigue cracks are also generated at the right end of the A rivet and rapidly expand to the right boundary. After the fatigue crack extends to rivet B, at this time, after a certain period of fatigue cycle, the crack on the right side of rivet B initiates and then extends to rivet C, and the crack propagation gradually accelerates. When the crack extends to rivet D, the crack propagates unsteadily. The photos of fatigue crack initiation and the crack propagation path of the No. 1 test piece are shown in Figure 15.

**Figure 16:**
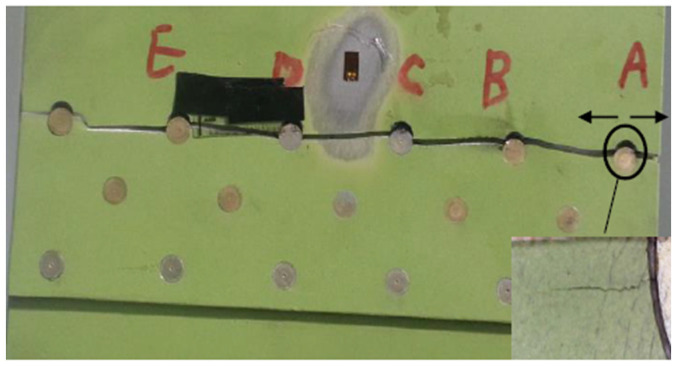
Fatigue crack initiation and fracture path of No. 2 test piece.

The fatigue crack of the No. 2 test piece initiates from the boundary of the A rivet on the top right of the back of the test piece and expands to the B rivet. When it expands to about 5 mm to the left, fatigue cracks are also generated at the right end of the A rivet and rapidly expand to the right boundary. After the fatigue crack extends to rivet B, at this time, after a certain cycle of fatigue cycle, the crack initiates on the left side of rivet B and then extends to rivet C, the crack propagation gradually accelerates and then extends in turn until the rivet E, the test piece breaks, and the test is completed. The photos of fatigue crack initiation and fracture path of the No. 2 test piece are shown in Figure 16.

In summary, the crack of the No. 1 test piece and No. 2 test piece initiates from A near the inner side and expands to B. When the crack expands to about 5 mm, the crack is generated on the other side of the hole A and rapidly expands to the plate boundary. In this paper, it is mainly considered that the crack of the No. 1 and No. 2 test pieces initiates a 1.5 mm crack inside hole A and expands to hole B, and the damage accumulation outside hole A is considered.

#### 4.2.3. Finite Element Simulation of Test Piece

When the fatigue crack propagation simulation analysis is carried out on the whole test piece model, the calculation data are too large. Therefore, the detailed analysis area of the test piece is selected for fatigue crack propagation simulation, and the initial crack length is 1.5 mm. Crack propagation is simulated in two stages.

The first stage: The initial crack at the right end of hole A is 1.5 mm. When the crack extends to a certain length, the damage accumulation on the left side of hole A reaches a critical value. Due to the randomness of damage in engineering practice, the initial damage on the left side of hole A is ignored in the simulation process of this paper. It is assumed that the initial damage is 0 and the critical damage is 1.

The second stage: The crack initiates on the left side of hole A and penetrates to the boundary, then expands to the right side with a new initial crack.

**Figure 17:**
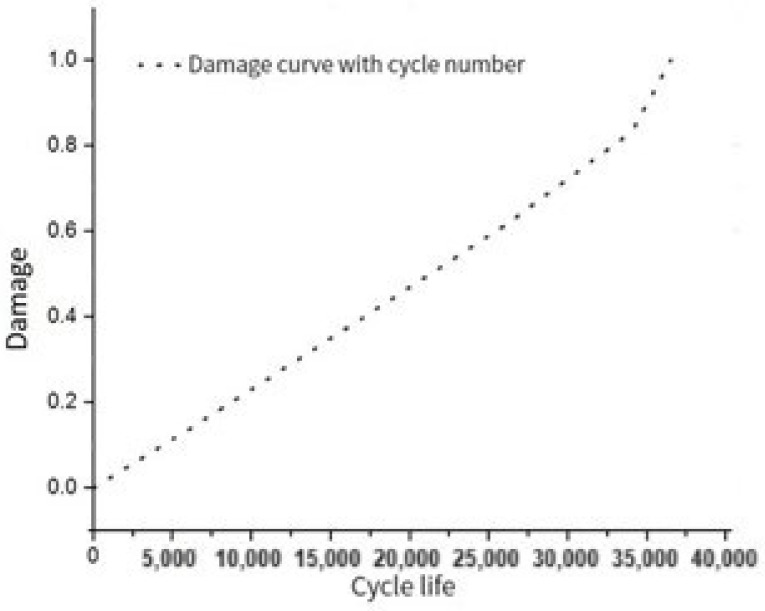
The curve of the left side injury of hole A with the number of cycles.

The first stage of finite element simulation of crack propagation: When the crack on the right side of the hole expands from the initial crack 1.5 mm to 5.36 mm, the damage value on the left side of hole A reaches the critical value D = 1 and the number of cycles N = 36,532. The curve of the damage on the left side of hole A with the number of cycles is shown in Figure 17.

**Figure 18:**
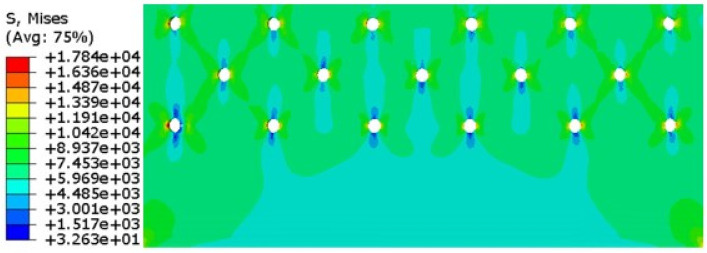
Finite element simulation of the stress cloud of the first stage of crack propagation.

When the crack extends to 1.8 mm, the number of cycles is N = 5835, and the stress cloud diagram is shown in Figure 18.

The second stage of finite element simulation of crack propagation: The crack penetrates the left boundary; when the crack on the right side of hole A extends to 23.75 mm (the actual crack length is 35.75 mm) and the crack propagation life N = 75,753 times, the crack is unstable and expands rapidly to the left side of hole B.

**Figure 19:**
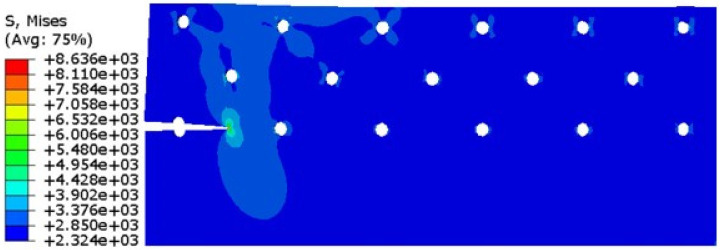
The stress cloud when the crack on the right side of hole A extends to 13 mm.

Among them, when the crack on the right side of hole A expands to 13 mm (the actual crack length is 25 mm), the number of cycles N = 58,946, the stress cloud diagram and local amplification are shown in Figure 19.

#### 4.2.4. Finite Element Simulation and Test Results Analysis

**Figure 20:**
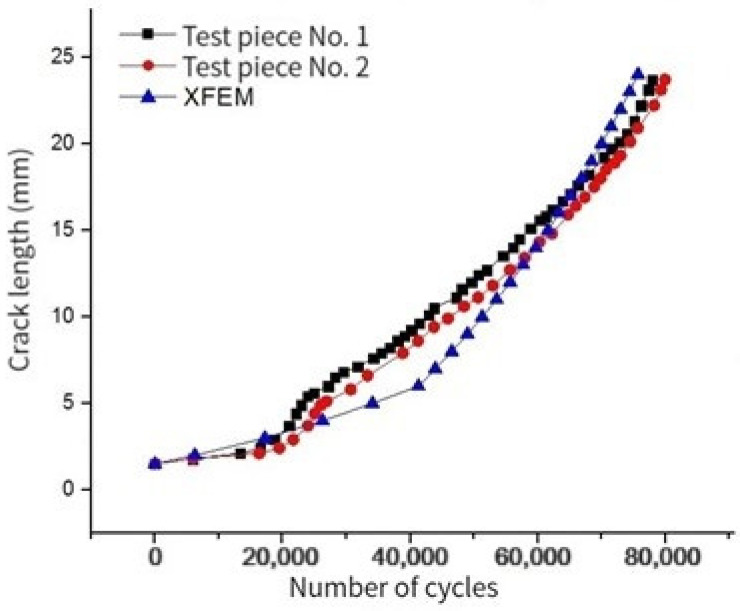
Comparison of XFEM simulation data and experimental data of crack propagation life.

In order to consider the damage accumulation on the left side of hole A, the crack propagation in the AB section is simulated. The comparison between the XFEM simulation data and the experimental data is shown in Figure 20.

It can be seen from the above figure that the growth trend of finite element simulation and experimental data is basically the same. In the early stage of crack propagation (initial crack on the left side of hole A), the number of cycles increases at a stable rate with crack growth. In the later stage of crack propagation (crack penetrates to the boundary), the initial crack length increases by 12 mm, and the change rate of the number of cycles with the crack length is completely different from that in the early stage. Then, the crack propagates stably at another rate until the crack propagates to 23.75 mm (the actual crack length is 35.75 mm). The crack is unstable and propagates rapidly to crack B.

By comparing the finite element simulation with the fatigue damage test data, we see that the error between the final simulation results and the test data is 3%. Because the initial damage on the left side of hole A is not considered in this paper, the number of cycles in the initial stage of crack propagation is larger than the experimental data. In addition, the life difference between the finite element simulation and the experimental data is N = 13,000 times when the crack propagation is 5.36 mm, and the actual error is about 13,010/77,945 = 16%, which is within the allowable error range of the project. In order to ensure safety, in the simulation test, a more conservative and higher safety factor simulation method is usually used to ensure that the life obtained by the physical test results is 10% to 20% larger than the simulation results. The simulation method in this project uses the conservative Walker formula, and the crack propagation rate formula does not consider the crack hysteresis effect. From the error comparison, we find that the method meets the design requirements of the simulation.

## 5. Summary

An extended finite element simulation analysis of a thin plate structure with a single eccentric hole, taking into account damage accumulation during crack propagation, was conducted. This analysis establishes a foundation for simulating fatigue damage tolerance in porous skin structures.

Furthermore, the fatigue damage tolerance test of the porous skin structure was simulated using a more conservative theoretical algorithm. When compared with the test results, the actual error is approximately 16%, meeting the requirements for engineering accuracy. The validity of the simulation program effectively addresses the issue of fatigue life in porous structures, from zero initial damage to structural failure, through extended finite element simulation.

## Figures and Tables

**Table 1:** Comparison of stress intensity factor results.

Crack Length and Proportion		$K/(\mathrm{MPa}\sqrt{mm})$		Error Ratio
a (mm)	a/W	XFEM	Theoretical Calculation	
8	0.1	6.02	5.86	2.7%
16	0.2	10.32	10.41	0.8%

**Table 2:** LY12-CZ material properties.

Parameters	Values
C	$4.5\times 10^{-11}$
n	3.41
KIC/$\;\mathrm{MPa}\sqrt{m}$	23.2
Kth0/$\;\mathrm{MPa}\sqrt{m}$	2.76
E/GPa	68
μ	0.33
$\sigma_{b}$/MPa	443
$\sigma_{ys}$/MPa	322

**Table 3:** The crack length at hole A and the damage at hole B under different cycles.

Total Number of Cycles N	Hole A Crack Length	Hole B Damage D
10,356	6 mm	2.78 × 10^−2^
40,875	10 mm	1.25 × 10^−1^
59,693	16 mm	2.09 × 10^−1^
65,861	20 mm	2.52 × 10^−1^
68,628	23 mm	2.93 × 10^−1^

**Table 4:** Test loading table.

Test Piece	Crack Initiation Situation	Peak–Valley Value of Load/KN
The first test piece	A (right) hole edge appears: 1.6 mm fatigue crack.	12.369/0.84
The second test piece	A (left) hole edge appears: 1.5 mm fatigue crack.	12.369/0.84

## Data Availability

Data available on request from the authors.

## References

[1] Li Z., Xu W., Zhang X. (2018). Experimental and analytical analyses of fatigue crack growth in sheets with multiple holes and cracks. Acta Aeronuatica Astronaut. Sin..

[2] Liu Y.J., Yue Z.F., Shao X.J., Liu J., Liu Y.S. (2009). Stress Intensity Factors for Multi-fastener-hole Structure with Multi-through-cracks. J. Mech. Strength.

[3] Sun H.B., Dong D.K. Computionl Study on the Boundary Correction Factor of Crack SIF in Porous Structure. Proceedings of the 27th China Conference on Structural Engineering.

[4] Hwu C., Huang S.-T., Li C.-C. (2017). Boundary-Based Finite Element Method for Two-Dimensional Anisotropic Elastic Solids with Multiple Holes and Cracks. Eng. Anal. Bound. Elem..

[5] Galatolo R., Lazzeri R. (2018). Fatigue Crack Growth of Multiple Interacting Cracks: Analytical Models and Experimental Validation. Fatigue Fract. Eng. Mater. Struct..

[6] Zou J., Yuechao Z., Feng Z. (2022). Multiple Crack Growth Simulation for Lap-Joints Based on Three-Dimensional Finite Element Analysis. Aircr. Eng. Aerosp. Technol..

[7] Theilig H., Goth M., Wünsche M. (2006). Numerical Simulation of Fatigue Crack Growth for Curved Cracks Emanating from Fastener Holes in Sheets by the MVCCI Method. Key Eng. Mater..

[8] Rao Q., Zhao C., Yi W., Sun D., Liu Z. (2022). Stress Intensity Factor Calculation of the Cracks Interacted by the Oval-Holes in Anisotropic Elastic Solids under Remote and Non-Uniform Surface Stresses. Theor. Appl. Fract. Mech..

[9] Cavuoto R., Lenarda P., Misseroni D., Paggi M., Bigoni D. (2022). Failure through Crack Propagation in Components with Holes and Notches: An Experimental Assessment of the Phase Field Model. Int. J. Solids Struct..

[10] Miao C., Yan X. (2013). Interaction of Three Parallel Cracks in Rectangular Plate under Cyclic Loads. Acta Mech. Solida Sin..

[11] Zhao J.F., Xie L.Y., Liu J.Z., Zhao Q. (2010). Modified Analytical Method for Stress Intensity Factor Calculation of Infinite MSD Plate Containing Multiple Holes. Adv. Mater. Res..

[12] Cao G., Liu X., Hu D., Mao J., Tian T., Wang R. (2023). Stochastic Modeling of Fatigue Crack Growth for Bolt Holes in Turbine Disc. Int. J. Fatigue.

[13] Seifi R., Ghadimian O., Ranjbaran M. (2015). Study on Life and Path of Fatigue Cracks in Multiple Site Damage Plates. Int. J. Fatigue.

[14] Wang W., Rans C., Benedictus R. (2018). Theoretical Analysis of Fatigue Failure in Mechanically Fastened Fibre Metal Laminate Joints Containing Multiple Cracks. Eng. Fail. Anal..

[15] Cui Y., Gao Y.F., Chew H.B. (2020). Two-Scale Porosity Effects on Cohesive Crack Growth in a Ductile Media. Int. J. Solids Struct..

[16] Chang P.-Y., Yeh P.-C., Yang J.-M. (2012). Fatigue Crack Growth in Fibre Metal Laminates with Multiple Open Holes: Fatigue Crack Growth in FMLs with Multiple Open Holes. Fatigue Fract. Eng. Mater. Struct..

[17] Ambriz R.R., García C., Rodríguez-Reyna S.L., Ramos-Azpeitia M., Jaramillo D. (2020). Synergy Effects in the Fatigue Crack Growth of Hole Cold Expanded Specimens under Variable Cyclic Loading. Int. J. Fatigue.

[18] Doan D.H., Zenkour A.M., Van Thom D. (2022). Finite Element Modeling of Free Vibration of Cracked Nanoplates with Flexoelectric Effects. Eur. Phys. J. Plus.

[19] Cong P.H., Van Thom D., Duc D.H. (2022). Phase Field Model for Fracture Based on Modified Couple Stress. Eng. Fract. Mech..

[20] Truong Anh T., Do Van T., Pham Tien D., Dinh Duc N. (2019). The Effects of Strength Models in Numerical Study of Metal Plate Destruction by Contact Explosive Charge. Mech. Adv. Mater. Struct..

[21] Van Do T., Hong Doan D., Chi Tho N., Dinh Duc N. (2022). Thermal Buckling Analysis of Cracked Functionally Graded Plates. Int. J. Struct. Stab. Dyn..

[22] Wang L., Zhang C., Tao C., Ji H., Yang Y., Qiu J. (2023). Prediction of Multiple Fatigue Crack Growth Based on Modified Paris Model with Particle Filtering Framework. Mech. Syst. Signal Process..

[23] Lv C., Wang F., Yang S., Zhao X. (2023). Studying a Repair Method of LY12 Aluminum Alloy Plate. Metals.

[24] Nguyen V.T., Hwu C. (2018). Multiple Holes, Cracks, and Inclusions in Anisotropic Viscoelastic Solids. Mech. Time-Depend. Mater..

[25] Xu W., Wu X.R., Yu Y. (2017). Weight Function, Stress Intensity Factor and Crack Opening Displacement Solutions to Periodic Collinear Edge Hole Cracks. Fatigue Fract. Eng. Mater. Struct..

[26] Anasori B., Saillot F., Stanley D., Awerbuch J., Tan T.-M. (2014). Fatigue Crack Growth in Aluminum Lithium Riveted Lap Joints. Procedia Eng..

[27] Fu Z., Ji B., Xie S., Liu T. (2016). Research on Multi-hole Arrangement in Stop-hole Technology for Retarding Fatigue Cracks in Steel Bridges. J. Hefei Univ. Technol. (Nat. Sci.).

[28] Yao Y., Ji B., Fu Z., Zhou J., Wang Y. (2019). Optimization of Stop-Hole Parameters for Cracks at Diaphragm-to-Rib Weld in Steel Bridges. J. Constr. Steel Res..

[29] Malekan M., Carvalho H. (2018). Analysis of a Main Fatigue Crack Interaction with Multiple Micro-Cracks/Voids in a Compact Tension Specimen Repaired by Stop-Hole Technique. J. Strain Anal. Eng. Des..

[30] Ahmed T., Yavuz A., Turkmen H.S. (2021). Fatigue Crack Growth Simulation of Interacting Multiple Cracks in Perforated Plates with Multiple Holes Using Boundary Cracklet Method. Fatigue Fract. Eng. Mater. Struct..

[31] Bhatia G.S., Arockiarajan A. (2022). Effect of Interactions of Two Holes on Tensile Behavior of Patch Repaired Carbon/Epoxy Woven Laminates. Def. Technol..

